# Tryptophan in the mouse diet is essential for embryo implantation and decidualization

**DOI:** 10.3389/fendo.2024.1356914

**Published:** 2024-05-01

**Authors:** Si-Ting Chen, Feng Ran, Wen-Wen Shi, Cheng-Kan Liu, Peng-Chao Wang, Hui-Na Luo, Zeng-Ming Yang

**Affiliations:** ^1^ College of Veterinary Medicine, South China Agricultural University, Guangzhou, China; ^2^ Key Laboratory of Animal Genetics, Breeding and Reproduction in the Plateau Mountain Region, College of Animal Science, Guizhou University, Guiyang, China; ^3^ College of Veterinary Medicine, Shanxi Agricultural University, Jinzhong, China

**Keywords:** tryptophan deficiency, decidualization, uterine receptivity, aryl hydrocarbon receptor, uterus

## Abstract

**Introduction:**

Nutritional deficiency occurs frequently during pregnancy and breastfeeding. Tryptophan (Trp), an essential amino acid which is critical for protein synthesis, serves as the precursor for serotonin, melatonin, and kynurenine (Kyn). The imbalance between serotonin and kynurenine pathways in Trp metabolism is closely related to inflammation and depression. This study assessed the effects of Trp deficiency on mouse early pregnancy.

**Methods:**

Embryo implantation and decidualization were analyzed after female mice had been fed diets containing 0.2% Trp (for the control group), 0.062% Trp (for the low Trp group) and 0% Trp (for the Trp-free group) for two months. The uteri of the mice were collected on days 4, 5, and 8 of pregnancy for further analysis.

**Results:**

On day 8 of pregnancy, the number of implantation sites were found to be similar between the control and the low Trp groups. However, no implantation sites were detected in the Trp-free group. On day 5 of pregnancy, plane polarity- and decidualization-related molecules showed abnormal expression pattern in the Trp-free group. On day 4 of pregnancy, there was no significant difference in uterine receptivity molecules between the low-Trp group and the control group, but uterine receptivity was abnormal in the Trp-free group. At implantation sites of the Trp-free group, IDO and AHR levels were markedly elevated. This potentially increased levels of Kyn, 2-hydroxy estradiol, and 4-hydroxy estradiol to affect decidualization.

**Conclusions:**

Trp-free diet may impair decidualization via the IDO-KYN-AHR pathway.

## Graphical Abstract

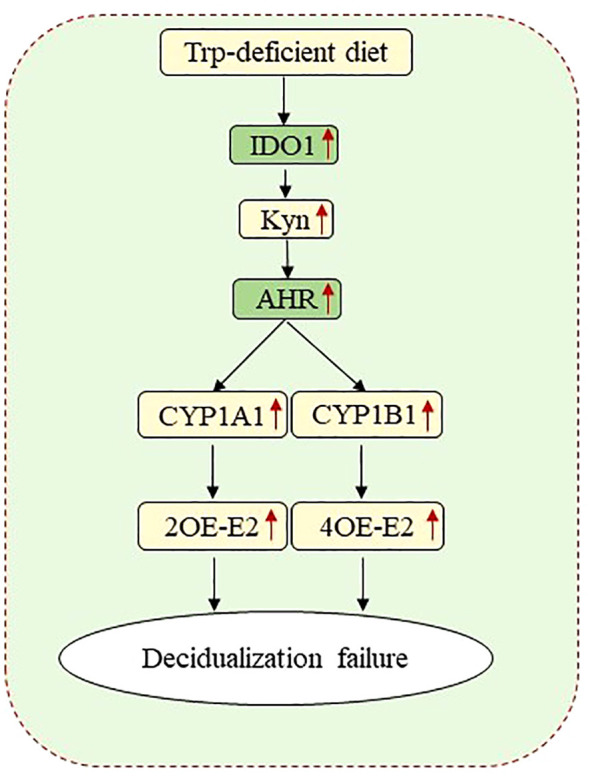

## 1 Introduction

Embryo implantation involves the interaction between the active blastocyst and a receptive uterus, and this even occurs only when the uterine environment is favorable for blastocyst implantation. Decidualization is the process by which endometrial stromal fibroblasts differentiate into decidual cells. Both embryo implantation and decidualization are essential for the establishment and maintenance of pregnancy in rodents and primates (1, 2).

Essential amino acids are required for pregnancy. Poor placental amino acid transport results in reduced fetal growth (3, 4). Tryptophan (Trp), an essential amino acid, can be only obtained through the diet. Adults need 3.5 mg of L-Trp/kg body weight/day to maintain nitrogen balance (5). The amount of Trp in corn and sorghum is low, so sufficient Trp is often lacking in poor areas or areas where corn and sorghum are staple foods (6, 7). Under-absorption of Trp can also cause Trp deficiency in Hartnup disease which causes under-absorption of neutral amino acids (including Trp) in the renal tubules and malabsorption of these amino acids in the gastrointestinal tract (8). Crohn’s disease is also associated with insufficient Trp absorption (9). Trp and its metabolites are involved in the production of environmental pollutants and affect body development (10, 11). Air pollutants may cause Trp metabolic disorders through oxidative stress and inflammation (12). Due to the increased needs of both females and fetuses for growth and development, Trp is particularly crucial during pregnancy (13, 14). Although Trp can be metabolized through the kynurenine (Kyn), 5-hydroxytryptamine, and indole pathways, more than 95% of Trp is metabolized via the Kyn pathway. The main rate-limiting enzymes in the Kyn pathway are Trp-2,3-dioxygenase (TDO), indoleamine-2,3-dioxygenase 1 (IDO1), and idoleamine-2,3-dioxygenase 2 (IDO2) (15, 16). IDO is essential for pregnancy because inhibition of IDO results in fetal loss in pregnant mice (17). TDO2 is highly expressed in mouse decidua. Inhibition of TDO2 leads to decreased expression of Cox2 and Vegf in decidual cells (18). Trp hydroxylase (TPH) can convert Trp to serotonin, a crucial neurotransmitter involved in the control of adaptive responses and reactions to environmental changes (19). Pups of Tph1-deficient mice have major developmental abnormalities in the brain and other tissues (20).

Dioxins and similar compounds are chlorinated organic pollutants that affect cell proliferation and differentiation by binding to the aryl hydrocarbon receptor (AHR). These chemicals also stimulate tumor growth and cause strong immunological, developmental, and reproductive damage through mechanisms unrelated to cytotoxicity (21, 22). Trp metabolites can bind to AHR (23). Ahr-/- mice have an impaired ability to support embryo-fetal development (24). Abnormal activation of AHR and the aryl hydrocarbon receptor nuclear translocator (ARNT) is common in endometriosis and uterine leiomyoma (25).

For growth and reproduction, the National Research Council recommended a dietary Trp dose of 2.0 g/kg for mice (16). The data from studies of diets containing high levels of Trp are contradictory. Although one study reported that L- Trp at 3.3 g/kg body weight/day during mouse pregnancy has no obvious effect on fetal growth (26), another study showed that a high L- Trp diet leads to a lower fetal weight (27). A Trp-deficient diet can prolong the lifespan by slowing physiological ageing (28, 29). Maternal Trp deficiency is associated with adverse offspring outcomes (17, 30). However, the effects of Trp deficiency on embryo implantation and decidualization remain to be defined.

Therefore, female mice were fed Trp-deficient, low-Trp or control diets for two months to evaluate the impact of these conditions on implantation and decidualization. The findings demonstrated that implantation and decidualization continued to occur normally even when the Trp deficit level reached 0.062%. Meanwhile, we found that a Trp -free diet can activate AHR and that a Trp-deficient diet results in pregnancy failure through activation of the IDO-KYN-AHR pathway in mice.

## 2 Results

### 2.1 Complete deficiency of Trp results in abnormal decidualization

**Figure 1:**
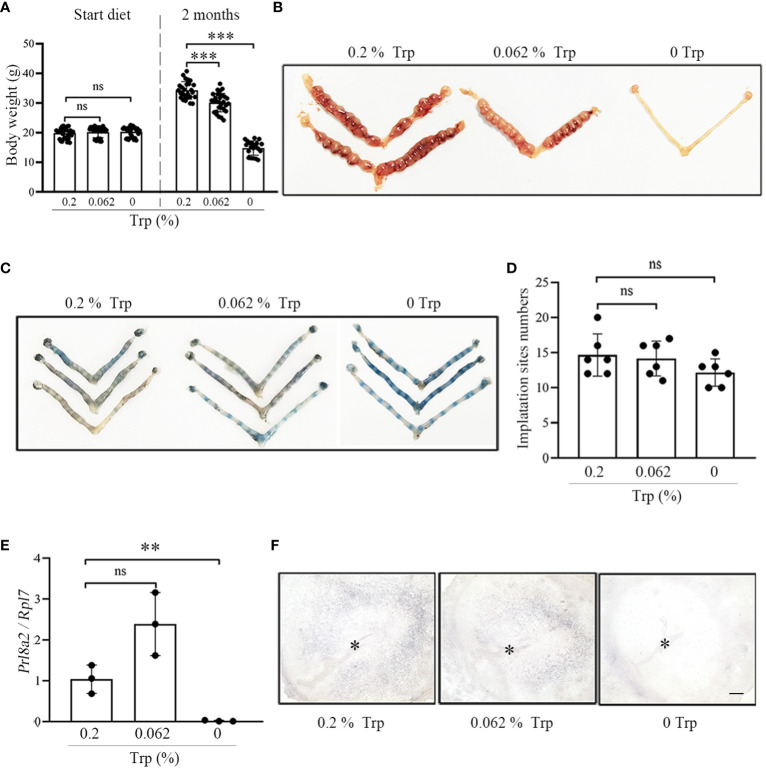
Complete Trp deficiency results in abnormal decidualization. **(A)** The body weight of female mice after feeding with different diet for two months. **(B)** A representative photograph showing the number of implantation sites on day 8 of pregnancy in each group (N=5 mice). **(C)** A representative photograph showing the number of implantation sites on day 5 of pregnancy in each group (N=5 mice). **(D)** Statistical analysis of the number of implantation sites on day 5 of pregnancy in each group. **(E)** qPCR analysis of Prl8a2 mRNA level in mouse uteri at implantation sites on day 5 in each group. **(F)** Alkaline phosphatase staining in mouse uteri on day 5 of implantation sites in each group. * Embryo. Scale bar, 50 μm. **, p < 0.01; ***, p < 0.001, ns, not significant.

The body weights of mice in the control group, low-Trp group and Trp-free group were comparable at the beginning of feeding (**Figure 1A**). The body weights of mice in the control group were significantly higher than that those of mice in the low-Trp and Trp-free groups after 2 months of feeding (**Figure 1A**).

To investigate the effects of Trp deficiency on early pregnancy, we examined mouse uteri in each group on day 8 of pregnancy. There was no obvious difference in the implantation sites between the low-Trp group and the control group on day 8 of pregnancy. In the Trp-free group, no implantation sites were observed in the uterus (**Figure 1B**). To explore the cause of pregnancy abnormalities on day 8 of pregnancy in the Trp-free group, we analyzed mouse uteri on day 5 of pregnancy. However, implantation sites were identified in each group, and the numbers of implantation sites were similar among the three groups (**Figures 1C, D**). We further examined the levels of decidualization-related markers.

Decidualization is critical for establishing pregnancy in mice (2). The prolactin family 8 subfamily A member 2 (Prl8a2) is a reliable marker for *in vitro* mouse decidualization (31). Compared with that in the control group, the expression of Prl8a2 in the low-Trp group was significantly higher, while Prl8a2 expression in the Trp-free group was significantly lower (**Figure 1E**). An increase in ALP activity is a marker for mouse decidualization (32). The density of alkaline phosphatase staining in the control group was higher than that in the low-Trp group, while alkaline phosphatase staining was not observed in the Trp-free group (**Figure 1F**). These results suggest that there was a decidualization abnormality in the Trp-free group.

### 2.2 Implantation chamber and planar cell polarity

**Figure 2:**
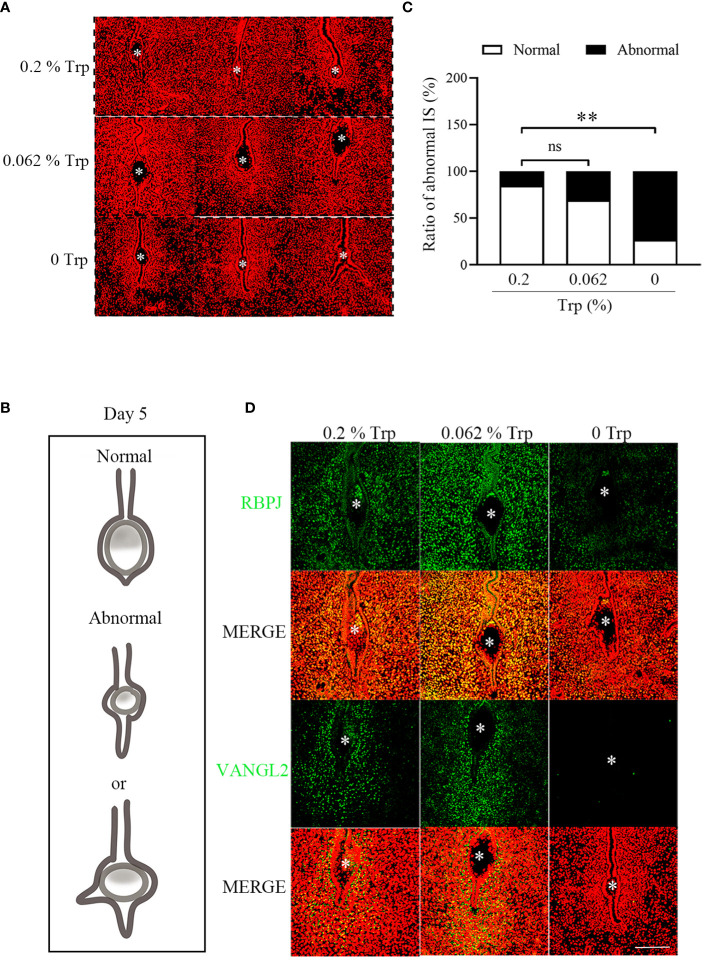
The morphology of implantation chambers and planar cell polarity signaling on day 5 of pregnancy. **(A)** The morphology of implantation chambers in each group. There were abnormal luminal closure and increased epithelial branching in Trp-free group. **(B)** A diagram showing normal and abnormal implantation chamber at implantation sites. **(C)** The number of abnormal implantation chambers and statistical analysis by the chi-square test (N=15 mice). IS: implantation sites. **(D)** Immunofluorescence of RBPJ and VANGL2 at implantation sites. * Embryo. Scale bar, 50 μm. **, p < 0.01, ns, not significant.

A unique crypt (implantation chamber) is established when luminal epithelial evaginations move towards the antimesometrial pole and are usually “spear shaped” (33, 34). On day 5 of pregnancy, the morphology of the implantation chamber was similar between the control group and the low-Trp group. Compared to that in the control group, the morphology of the implantation chamber in the Trp-free group was abnormal. There were significantly more abnormal implantation chambers in the Trp-free group than in both the control and low-Trp groups (**Figures 2A–C**).

Planar cell polarity (PCP) signaling is necessary for crypt development (35). Van Gogh-Like Protein 2 (VANGL2) and Rbpsuh (RBPJ) are key PCP components. Epithelial Vangl2 null mice exhibit shallower luminal epithelial eversions and defects in crypt shape and size (36). In Rbpj-deficient mice, a seriously deviated uterus embryo axis at the implantation site, abnormal decidual morphology, and impaired embryonic development were observed (37). Therefore, we examined the expression of PCP signaling molecules in the uterus on day 5 of pregnancy in each group. The immunofluorescence intensity of VANGL2 and RBPJ in the uterus of the Trp-free group was lower than that in the control group, while the immunofluorescence signal in the low-Trp group was slightly greater than that in the control group (**Figure 2D**). These results suggest that PCP signaling is impaired in the Trp-free group.

### 2.3 Uterine receptivity

**Figure 3:**
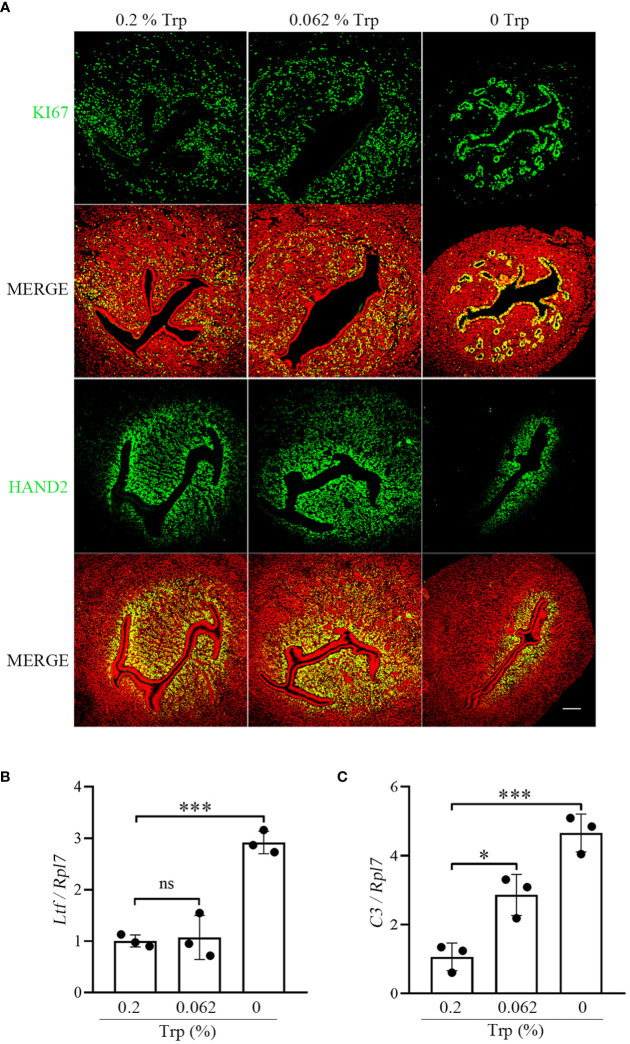
Uterine receptivity on day 4 of pregnancy. **(A)** Ki67 immunostaining and HAND2 immunofluorescence. * Embryo. Scale bar, 50 μm. **(B)** qPCR analysis of *Ltf* mRNA level. **(C)** qPCR analysis of *C3* mRNA level. *, p < 0.05; ***, p < 0.001; ns, not significant.

A successful pregnancy requires proper interaction between the activated embryo and the receptive uterus. On day 4 of pregnancy, uterine epithelial cells stop proliferating and begin to differentiate into the receptive phase (38, 39). Compared to that in the control group, immunostaining for Ki67, a marker of cell proliferation, was increased in epithelial cells on day 4 of pregnancy in the Trp-free group, while Ki67 immunostaining in the low-Trp group was similar to that in the control group (**Figure 3A**). Compared to that in the control and low-Trp groups, the number of subluminal stromal cells immunostained for Hand2 was obviously lower in the Trp-free group (**Figure 3A**). Lactoferrin (LTF) and complement C3 (C3) are target genes of oestrogen signaling (40, 41). Compared to that in the control group, Ltf and C3 expression in the Trp-free group was significantly higher. In the low-Trp group, the expression of Ltf was similar to that in the control group, but C3 expression was significantly higher than that in the control group (**Figures 3B, C**). Suppression of oestrogen-mediated uterine epithelial proliferation by progesterone is a prerequisite for successful implantation. HAND2, a progesterone-stimulated gene, is expressed in the uterine stroma during the peri-implantation period and is essential for mouse embryo implantation (39). These results suggest that a Trp-free diet for two months might have adverse effects on uterine receptivity.

### 2.4 The IDO-kynurenine pathway of Trp metabolism at the implantation site

**Figure 4:**
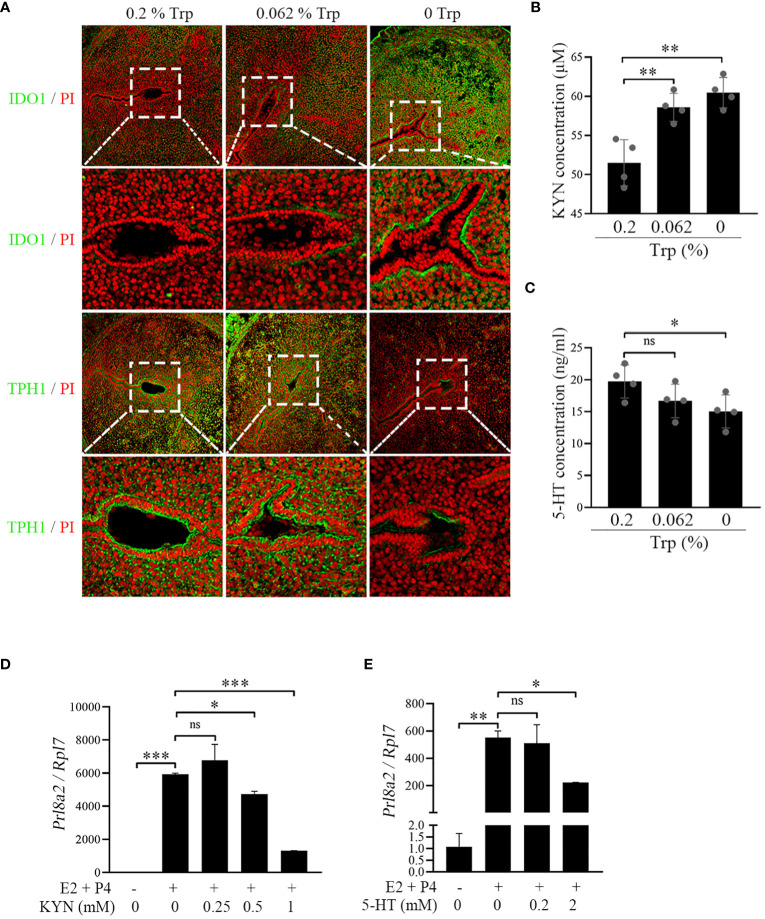
IDO-kynurenine pathway at implantation site on day 5 of pregnancy. **(A)** Immunofluorescence of IDO1 and TPH1. * Embryo. Scale bar, 50 μm. **(B)** Uterine Kyn concentration at implantation site. **(C)** Uterine 5-HT concentration at implantation site. **(D)** qPCR analysis of *Prl8a2* mRNA level after mouse stromal cells were treated with Kyn for 48 h under *in vitro* decidualization. **(E)** qPCR analysis of *Prl8a2* mRNA level after mouse stromal cells were treated with 5-HT for 48 h under *in vitro* decidualization. *, p < 0.05; **, p < 0.01; ***, p < 0.001, ns, not significant.

Although Trp is metabolized to Kyn mainly via IDO1 and Tdo2, Trp is also used to synthesize serotonin via the TPH1 enzyme (13). To further explore the reasons for the adverse effects of Trp deficiency on early pregnancy, we examined the key enzymes and their products involved in Trp metabolism. Compared to those in the control and low-Trp groups, there was an increase in IDO1 immunofluorescence and a decrease in TPH1 immunofluorescence in the Trp-free group (**Figure 4A**). The uterine Kyn concentrations in the Trp-free and low-Trp groups were significantly higher than those in the control group (**Figure 4B**). However, the uterine serotonin concentration in the Trp-free group was significantly lower than that in the control and low-Trp groups (**Figure 4C**). These data suggest that Trp is metabolized mainly into Kyn. Under *in vitro* decidualization, the expression of *Prl8a2*, a marker of mouse *in vitro* decidualization (42), was significantly increased, which was significantly inhibited by either 0.5 or 1 mM Kyn (**Figure 4D**). In addition, treatment of mouse stromal cells with 5-HT under *in vitro* decidualization conditions revealed that low concentrations of 5-HT had minimal effects on *Prl8a2* expression, while higher concentrations of 5-HT suppressed *Prl8a2* expression (**Figure 4E**). Therefore, the high uterine Kyn concentration resulting from strong IDO1 expression impaired mouse decidualization in the Trp-free group.

### 2.5 Kynurenine-induced AhR signaling

**Figure 5:**
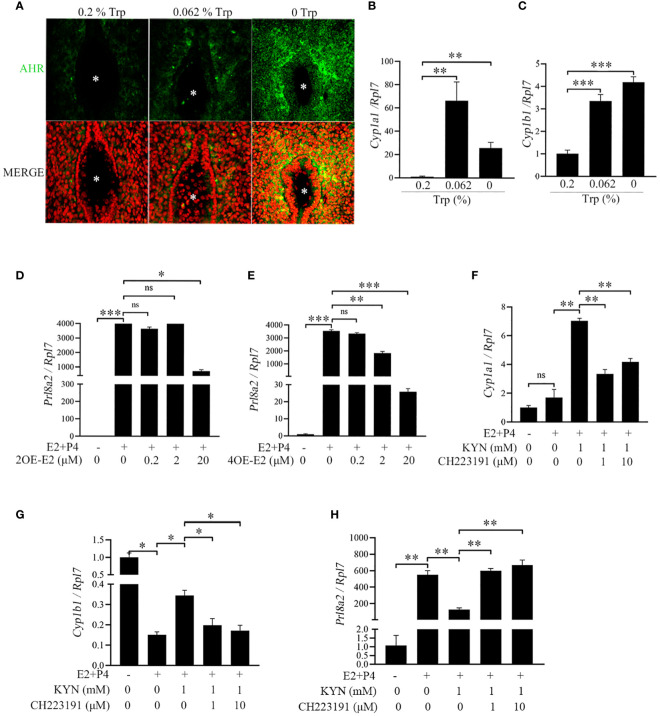
AHR signaling and its effects on mouse decidualization. **(A)** AhR immunofluorescence at implantation site on day 5 of pregnancy. * Embryo. Scale bar, 50 μm. **(B)** qPCR analysis of *Cyp1a1* mRNA level in mouse uterus at implantation sites on day 5 of pregnancy. **(C)** qPCR analysis of *Cyp1b1* mRNA level in mouse uterus at implantation site on day 5 of pregnancy. **(D)** qPCR analysis of *Prl8a2* mRNA level after mouse stromal cells were treated with 2OE-E2 for 48 h under *in vitro* decidualization. **(E)** qPCR analysis of *Prl8a2* mRNA level after mouse stromal cells were treated with 4OE-E2 for 48 h under *in vitro* decidualization. **(F)** qPCR analysis of *Cypla1* mRNA level after mouse stromal cells were treated with CH223191 (AHR inhibitor) for 48 h in the presence or absence of Kyn under *in vitro* decidualization. **(G)** qPCR analysis of *Cyplb1* mRNA level after mouse stromal cells were treated with CH223191 for 48 h in the presence or absence of Kyn under *in vitro* decidualization. **(H)** qPCR analysis of *Prl8a2* mRNA level after mouse stromal cells were treated with CH223191 for 48 h in the presence or absence of Kyn under *in vitro* decidualization. *, p < 0.05; **, p < 0.01; ***, p < 0.001, ns, not significant.

Because Trp and Kyn can activate AHR (43) and the AHR target genes CYP1A1 and CYP1B1 (44, 45), the expression of AHR and its target genes was examined. Compared with that in the low-Trp and control groups, AHR immunofluorescence was strongly detected in the subluminal stromal cells at the implantation site in the Trp-free group (**Figure 5A**). Additionally, the expression of CYP1A1 and CYP1B1 was significantly higher in the Trp-free and low-Trp groups than in the control group (**Figures 5B, C**). Cytochrome CYP1A1 and CYP1B1 catalyze the oxidative metabolism of oestradiol to produce the catechol oestrogens 2-hydroxy oestradiol (2OE-E2) and 4-hydroxy oestradiol (4OE-E2), respectively (46, 47). Under *in vitro* decidualization, *Prl8a2* expression was significantly increased, which was suppressed by 2OE-E2 (20 μM) and 4OE-E2 (2 and 20 μM) (**Figures 5D, E**). Treatment of mouse stromal cells with Kyn under *in vitro* decidualization significantly increased *Cyp1a1* and *Cyp1b1* expression. CH223191, an AHR inhibitor, abrogated Kyn-induced *Cyp1a1* and *Cyp1b1* upregulation (**Figures 5F, G**). Moreover, treatment of mouse stromal cells with Kyn under *in vitro* decidualization significantly downregulated *Prl8a2* expression, while CH223191 could improve the inhibition of decidualization by Kyn (**Figure 5H**). Taken together, these results suggest that activated AHR and downstream genes contribute to abnormal decidualization in the Trp-free group.

## 3 Discussion

In our study, mice fed a Trip-free diet for two months had abnormalities in uterine receptivity, the implantation chamber and decidualization. However, a diet in which the Trp content was reduced to 0.062% had little effect on early pregnancy. Trp is an essential amino acid necessary for protein synthesis and can be obtained only from the diet (48, 49). Although Trp deficiency has been extensively investigated in different animal species (50, 51), its effects on embryo implantation and decidualization are still unclear.

In our study, the diet containing 0.062% Trp had no obvious negative effects on uterine receptivity, implantation, or decidualization. A previous study showed that mice fed 0.08% Trp had greater survival and lower growth than did fully fed control mice (29). Symptoms of Trp insufficiency may manifest when the consumption level falls short by a mere 25% of the recommended intake. These symptoms include diminished food consumption, decelerated growth, compromised bone formation and aberrant behavior (52). Trp deficiency leads to an increase in climbing behavior in male rats but has no obvious effect on sexual behavior in female rats (53, 54). Maternal Trp deficiency is associated with adverse offspring outcomes, including decreased body weight and growth retardation (30, 55).

In our study, although implantation sites were detected on day 5 of pregnancy in the Trp-free group, no implantation sites were observed on day 8 of pregnancy. In the Trp-free group, most of the crypts at the implantation site had an abnormal “spear shape”. RBPJ and VANGL2 are two key components of the PCP signaling pathway that are essential for crypt formation (35). In our study, the levels of RBPJ and VANGL2 were obviously lower in the Trp-free group than in the control group. Because defects in crypt shape and size have adverse effects throughout pregnancy (36), abnormalities in PCP signaling may result in pregnancy failure.

Our results may indicate that early pregnancy in mice fed a Trp-free diet may be affected through the IDO-Kyn pathway. IDO is the major rate-limiting enzyme for metabolizing Trp to Kyn (56). In our study, IDO1 protein levels increased at the uterine implantation sites in mice fed a Trp-free diet and Kyn concentrations also increased significantly compared to control mice. During normal pregnancy, IDO1 expression was highest on day 4 of pregnancy and decreased on days 5-8 of pregnancy. IDO1 overexpression significantly decreased the expression of the decidualization marker Prl8a2 (57). In a human study, Kyn promoted decidualization of human endometrial stromal cells (58). However, mouse *in vitro* decidualization was significantly decreased by Kyn, which is opposite to what occurs during human decidualization. This difference may be attributed to differences in species and Kyn concentrations. Changes in kynurenine pathway metabolite concentrations may play a role in the pathophysiology of pregnancy complications (59). TPH1 is the first and rate-limiting step in serotonin biosynthesis (60). TPH1 protein levels were significantly reduced in mice fed a Trp-free diet than that in the control group, and the concentration of serotonin was also significantly lower in mice fed a Trp-free diet than that in the control group. Inducing serotonergic dysfunction in rats using a Trp-free diet is a quick, painless, efficient, and very precise way to reduce serum serotonin concentration (61). Moreover, Tph1-deficient female mice have abnormal central nervous system development (62). Adequate serotonin is essential for fetal development and contraction of the myometrium (63, 64).

Kyn may control decidualization via AHR. CYP1A1 and CYP1B1, two AhR target genes, catalyze the oxidative metabolism of oestradiol to produce 2OE-E2 and 4OE-E2, respectively. Both 2OE-E2 and 4OE-E2 can promote human *in vitro* decidualization (57). However, in our study, mouse *in vitro* decidualization was inhibited by either 2OE-E2 or 4OE-E2. Kyn, an endogenous agonist of AHR, controls several immunological and physiological processes (58). AHR plays an important role as a sensor of environmental pollutants and is closely related to multiple signaling pathways involved in early development and pathogenesis (65). AHR mediates the effects of many environmental endocrine disruptors and leads to the loss of normal ovarian function in polluted environments (66). Cyp1a1 and Cyp1b1 catalyze functional reactions in biological metabolism and are of toxicological importance (22). The AHR signaling cascade is involved in oestrogen regulation in the female reproductive system (67). On day 4 of pregnancy, Cyp1b1 mRNA was strongly expressed in subluminal stromal cells and weakly expressed in epithelial cells. Oestrogen may induce its own degradation of toxic 4OH E2 by increasing the expression of CYP1B1 in the uterus. Delayed implantation in mice can be initiated by 4OH E2 (68, 69). In conclusion, a diet devoid of Trp may impair decidualization via the IDO-KYN-AHR pathway.

## 4 Conclusion

In our study, a low-Trp diet (0.062% Trp) had no obvious impact on implantation or decidualization. However, a complete lack of Trp led to abnormal decidualization via the IDO-KYN-AHR pathway in mice.

## 5 Materials and methods

### 5.1 Animals and diet

Female CD-1 mice (6 weeks old) were bought from Hunan Slaike Jingda Laboratory Animal Co., Ltd. (Chaina, Hunan) and kept in a temperature controlled environment with a 12 h light photoperiod. The animals were given free food and water. All animal protocols were approved by the Animal Care and Use Committee of South China Agricultural University (No. 2021f085). Female mice were co-caged with fertile or vasectomized males to induce pregnancy or pseudopregnancy. The day when vaginal plug was observed was defined as day 1 of pregnancy. From days 1 to 4, pregnancy was confirmed by collecting embryos from oviducts or uterus. Implantation sites on days 5 and 6 of pregnancy were visualized by intravenous injection of 0.1 ml of 1% Chicago blue dye (Sigma-Aldrich). Artificial decidualization was induced by intraluminal injection of 10 μl of sesame oil (Sigma-Aldrich). The uninjected contralateral uterine horn served as control.

According to the nutrient requirements of laboratory animals, the Trp content in the maintenance feed for mice is 0.1%, and the Trp content in the breeding feed is 0.2% (70). In a previous study, Trp amount in a Trp-limited diet is 0.062% (52). Because we mainly studied the effects of Trp content on early pregnancy, female mice were fed different diets containing 0.2% Trp (for control group, N=35 mice), 0.062% Trp (for low-Trp group, N=35 mice) and 0% Trp (for Trp-free group, N=35 mice), respectively. Mouse diets containing different concentrations of Trp were purchased from Jiangsu Xietong Pharmaceutical Bio-engineering Co., Ltd. (Jiangsu, China),. All proteins in the mouse diets were replaced with amino acid premixes in the process of making feed to ensure that the Trp content in the food was 0.2%, 0.062% and 0 of the total feed (see **Table 1** for specific feed formulations). *In vivo* experiments with dietary for Trp addition or deficiency are generally a long-term process for 12 weeks, 8 weeks, or 70 days in previous studies (71–74). Therefore, we first fed female mice with different amounts of Trp for 2 months and then performed different treatments. Pregnant or pseudopregnant mice were also fed with the diets containing different concentrations of Trp until they were sacrificed by cervical dislocation. Female body weights were comparable in three groups at the beginning of feeding. The uteri and serum were collected on days 4, 5 and 8 of pregnancy for further analysis.

**Table 1:** Composition of diets.

	0 Trp	0.062% Trp	0.2% Trp (g/kg diet)
Corn Starch	552.5	551.88	550.5
Maltodextrin 10	125	125	125
Cellulose	50	50	50
Corn Oil	50	50	50
Mineral Mix S10001	35	35	35
Sodium Bicarbonate	7.5	7.5	7.5
Vitamin Mix V10001	10	10	10
Choline Bitrartrate	2	2	2
L-Tryptophan	0	0.62	2
**L-Methionine**	6	6	6
**L-Alanine**	10	10	10
**L-Arginine**	10	10	10
**L-Asparagine**	5	5	5
**L-Aspartate**	10	10	10
**L-Cystine**	4	4	4
**L-Glutamic Acid**	30	30	30
**L-Glutamine**	5	5	5
**Glycine**	10	10	10
**L-Histidine-HCl**	6	6	6
**L-Isoleucine**	8	8	8
**L-Leucine**	12	12	12
**L-Lysine-HCl**	14	14	14
**L-Phenylalanine**	8	8	8
**L-Proline**	5	5	5
**L-Serine**	5	5	5
**L-Threonine**	8	8	8
**L-Tyrosine**	4	4	4
**L-Valine**	8	8	8

### 5.2 Alkaline phosphatase staining

Alkaline phosphatase (ALP) staining was performed as previously described (75). Frozen sections (10 μm) of mouse uteri were quickly fixed for 10 min in 4% paraformaldehyde (158127, Sigma Aldrich, St. Louis, MO). After three washes in PBS, sections were incubated with nitro blue tetrazolium (0885, Life Science) and 5-bromo-4-chloro-3-indolyl phosphate (0329, Life Science, St. Louis, MO) in PBS solution for 10 min to show alkaline phosphatase activity. The staining density was examined under a microscope. Each experiment was repeated at least three times.

### 5.3 Immunofluorescence

The immunofluorescence method was performed as previously described (76, 77). Frozen sections (10 μm) were fixed with 4% paraformaldehyde (158127, Sigma Aldrich) for 10 min. Paraffin sections (5 μm) were dewaxed, hydrated and antigen-retrieved by boiling in 10 mM citric acid buffer for 10 min. After permeabilized with 0.1% Triton 100 and blocked with 10% horse serum at 37° C for 1 h, sections were incubated with appropriate dilutions of primary antibodies overnight at 4° C. Primary antibodies used in this study included anti-KI67 (1:200, GB111141, Exilon, Guangzhou), anti-RBPJ (1:200, 14613, Proteintech, Wuhan, China), anti-VANGL2 (1:200, 21492, Proteintech), anti-HAND2 (1:200, sc-9409, Santa Cruz Biotechnogoly, Santa Cruz, USA), anti-IDO1 (1:200, 66528, Proteintech), anti-TPH1 (1:100, CSB-PA024100LA01HU, Cusabio, Wuhan, China). After washing with PBS, sections were incubated with 488-conjugated secondary antibodies (2.5 μg/ml, G21234, Invitrogen, Carlsbad, CA) for 40 min, counterstained with propidium iodide (5 μg/ml, PI, P4170, Sigma-Aldrich) and mounted with Extended™ Diamond Antifade Mountant (Thermo Fisher, Waltham, MA). Images were taken with a laser scanning confocal microscope (Leica, Germany). Each experiment was repeated at least three times.

### 5.4 Kynurenine assay

Kyn concentration was determined as previously described (78). In short, after the mouse uterus was collected at implantation sites on day 5 of pregnancy, the endometrium was isolated from the myometrium and homogenized in sample diluent. After centrifugated at 5000×g for 10 min, a total of 360 μl supernatant was mixed with 180 μl of 30% trichloroacetic acid (TCA) (T6399, Sigma) and incubated at 50° C for 30 min. After centrifugation at 3000×g for 10 min, the supernatant was mixed with equal volume of current Ehrlich reagent (2% p-dimethylaminobenzaldehyde, D109644, Aladdin, Shanghai, China) dissolved in glacial acetic acid) thoroughly, and incubated at room temperature for 12-30 min. Absorption was measured at 492 nm and compared with the standard curve of L-Kyn (HY-104026, MedChemExpress, NJ, USA). Each experiment was repeated at least three times.

### 5.5 ELISA assay

After removing myometrium, the endometrium at implantation sites on day 5 of pregnancy was homogenized for serotonin measurement according to the manufacturer’s instructions (Elabscience, E-EL-0033c, Wuhan, China). This kit’s sensitivity is greater than 9.38 ng/ml. The standard working solution or samples of 50 μL were mixed with 50 μL biotinylated antibody in working solution and incubated at 37° C for 45 min. Following washing 3 times, 100 μL HRP enzyme conjugate working solution was added to each well and incubated at 37° C for 30 min. After washing 5 times, 90 μL of substrate solution was added to each well and incubated at 37° C for 15 min. Absorbance at 450 nm wavelength was measured following adding 50 μL of termination solution to each well. Each experiment was repeated at least three times.

### 5.6 Isolation and treatment of mouse endometrial stromal cells

Mouse endometrial stromal cells were isolated as previously mentioned (77). Mouse uteri on day 4 of pseudopregnancy were longitudinally cut, washed in HBSS, and incubated with 1% (W/V) trypsin and 6 mg/ml dispase in 3.5 mL HBSS for 1 h at 4°C, 1 h at room temperature, and 10 min at 37°C. The uterine tissues were washed with Hanks’ balanced salt solution and incubated with 6 ml of HBSS with 0.15 mg/ml Collagenase I (Invitrogen, 17100-017) at 37°C for 35 min. Primary endometrial stromal cells were cultured in DMEM/F12 with 10% FBS.

Mouse endometrial stromal cells were induced for *in vitro* decidualization as previously described (79). Endometrial stromal cells were treated with 10 nM of estradiol-17 β and 1 μM of P4 in DMEM/F12 containing 2% charcoal-treated FBS (cFBS, Biological Industries) to induce decidualization *in vitro* for 72 h. Stromal cells were treated with L- Kyn (0.25, 0.5 and 1 mM, HY-104026, MedChemExpress), 4-Hydroxyestradiol (0.2, 2 or 20 mM, GC19552, Glpbio, Shanghai, China), 2-Hydroxyestradiol (0.2, 2 or 20 mM, GC13460, Glpbio) in DMEM/F12 containing 2% cFBS, respectively. Each experiment was repeated at least three times.

### 5.7 Real-time RT-PCR

RT-qPCR was performed as previously described (80). Total RNAs were digested with RQ1 deoxyribonuclease I (Promega, Fitchburg, WI) to remove DNA, and reverse-transcribed into cDNA with Prime Script Reverse Transcriptase Reagent Kit (Takara, Japan). Real-time PCR was performed using a SYBR Premix Ex Taq Kit (TaKaRa) on the CFX96 TouchTM Real-Time System (Bio-Rad) The 2-△△Ct method was used to calculate the data, which were standardized to the RPL7 (mouse) or RPL7 (human) level. The primer sequences for each gene were provided in **Table 2**. Each experiment was repeated at least three times.

**Table 2:** Primers and siRNA sequences used in this study.

Gene	Species	Sequence (5’-3’)	Application	Accession Number	Product size
*Rpl7*	Mouse	GCAGATGTACCGCACTGAGATTC ACCTTTGGGCTTACTCCATTGATA	RT-qPCR	NM_011291.5	129 bp
*Prl8a2*	Mouse	AGCCAGAAATCACTGCCACT TGATCCATGCACCCATAAAA	RT-qPCR	NM_010088	119 bp
*Ltf*	Mouse	AGCCAACAAATGTGCCTCTTC CCTCAAATACCGTGCTTCCTC	RT-qPCR	NM_008522	119 bp
*C3*	Mouse	TGGACCAGACCGAACAGT GAAGGCAGCATAGGCAGA	RT-qPCR	NM_009778.3	125 bp
*Cyp1a1*	Mouse	CAGAAGGTGATGGCAGAG ACGGAGGACAGGAATGAA	RT-qPCR	NM_001136059.2	201 bp
*Cyp1b1*	Mouse	CTGGACTTGGAGGATGTG GCTGGAGAATCGCATTGA	RT-qPCR	NM_009994.2	237 bp

### 5.8 Statistical analysis

Data were processed using GraphPad Prism 8.0. The student’s t test was employed to compare the difference between the two groups. The ANOVA test was utilized to compare the difference of more than two groups. At least three independent replications of each experiment were conducted. Each group in the mouse study contained a minimum of three mice. Standard deviation (SD) and mean were used to present the data. A result with a p-value of 0.05 was deemed significant.

## Data availability statement

The original contributions presented in the study are included in the article/supplementary material. Further inquiries can be directed to the corresponding author.

## Ethics statement

The animal study was approved by Animal Care and Use Committee of South China Agricultural University. The study was conducted in accordance with the local legislation and institutional requirements.

## Author contributions

STC: Writing – review & editing, Data curation, Investigation, Validation, Writing – original draft. FR: Investigation, Writing – review & editing. WWS: Investigation, Writing – review & editing. CKL: Investigation, Writing – review & editing. PCW: Investigation, Writing – review & editing. HNL: Investigation, Writing – review & editing. ZMY: Writing – review & editing, Conceptualization, Funding acquisition, Project administration, Supervision.
